# Structure of the Diphtheria Toxin at Acidic pH: Implications for the Conformational Switching of the Translocation Domain

**DOI:** 10.3390/toxins12110704

**Published:** 2020-11-07

**Authors:** Mykola V. Rodnin, Maithri M. Kashipathy, Alexander Kyrychenko, Kevin P. Battaile, Scott Lovell, Alexey S. Ladokhin

**Affiliations:** 1 Department of Biochemistry and Molecular Biology, University of Kansas Medical Center, Kansas City, KS 66160, USA; mrodnin@kumc.edu (M.V.R.); alexander.v.kyrychenko@univer.kharkov.ua (A.K.); 2 Protein Structure Laboratory, Shankel Structural Biology Center, University of Kansas, Lawrence, KS 66047, USA; m_kashipathy@ku.edu (M.M.K.); swlovell@ku.edu (S.L.); 3 Institute of Chemistry and School of Chemistry, V. N. Karazin Kharkiv National University, 61022 Kharkiv, Ukraine; 4 NYX beamline, New York Structural Biology Center, Upton, NY 11973, USA; battaile@nysbc.org

**Keywords:** diphtheria toxin structure, X-ray crystallography, helix unfolding, acidification, conformational switching

## Abstract

Diphtheria toxin, an exotoxin secreted by *Corynebacterium* that causes disease in humans by inhibiting protein synthesis, enters the cell via receptor-mediated endocytosis. The subsequent endosomal acidification triggers a series of conformational changes, resulting in the refolding and membrane insertion of the translocation (T-)domain and ultimately leading to the translocation of the catalytic domain into the cytoplasm. Here, we use X-ray crystallography along with circular dichroism and fluorescence spectroscopy to gain insight into the mechanism of the early stages of pH-dependent conformational transition. For the first time, we present the high-resolution structure of the diphtheria toxin at a mildly acidic pH (5–6) and compare it to the structure at neutral pH (7). We demonstrate that neither catalytic nor receptor-binding domains change their structure upon this acidification, while the T-domain undergoes a conformational change that results in the unfolding of the TH2–3 helices. Surprisingly, the TH1 helix maintains its conformation in the crystal of the full-length toxin even at pH 5. This contrasts with the evidence from the new and previously published data, obtained by spectroscopic measurements and molecular dynamics computer simulations, which indicate the refolding of TH1 upon the acidification of the isolated T-domain. The overall results imply that the membrane interactions of the T-domain are critical in ensuring the proper conformational changes required for the preparation of the diphtheria toxin for the cellular entry.

## 1. Introduction

**Figure 1:**
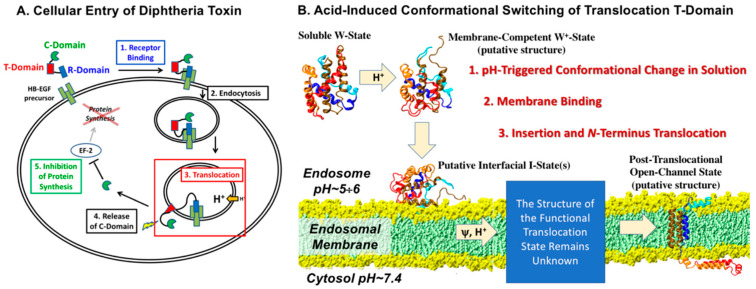
(**A**) Schematic representation of the cellular entry of diphtheria toxin (DT). (**B**) Summary of in vitro studies of the pH-triggered membrane insertion pathway of the diphtheria toxin T-domain, responsible for bridging the endosomal membrane (red square in **A**). Modified from [10,25]. The initial step of acid-induced conformational switching prior to membrane interactions is the subject of this study.

Diphtheria toxin (DT) is secreted by *Corynebacterium* and causes disease in humans by inhibiting protein synthesis. DT consists of three domains—receptor (R-), translocation (T-), and catalytic (C-)—and similar to many other A-B toxins it enters the cell via the endosomal pathway [1,2,3,4,5] (Figure 1a). After the binding of the R-domain to the EGFR-like receptor on the cell surface, the toxin is endocytosed into the cell, and acidification inside the endosome promotes the requisite conformational change and membrane insertion of the T-domain followed by the translocation of the C-domain into the cytosol. Following translocation, the C-domain is proteolyzed from the R and T domains and proceeds to inhibit the EF2 translation factor, leading to the termination of protein synthesis and cell death. The DT T-domain translocates the catalytic domain across the endosomal membrane without the help of any additional protein components [6] and apparently does so as a monomer [7,8]. The T-domain can also translocate other proteins in a pH-dependent manner, provided they form a molten globule-like state [9].

A schematic representation of the pH-dependent membrane insertion pathway of the T-domain is shown in Figure 1b [10]. The protonation of key histidine residues is involved in the formation of the membrane-competent W^+^-state [11,12,13], which rapidly associates with the bilayer to form an interfacial intermediate I-state [10]. Subsequent insertion is facilitated by the presence of anionic lipids, which decrease the thermodynamic barrier for the insertion. The two protonation steps responsible for the formation of conformations capable of membrane association and insertion have overlapping pH ranges, suggesting that additional protonation can occur at the same pH value due to the shift in the pK_a_ values of titratable residues after their partitioning into the interfacial zone of the lipid bilayer. While numerous studies have shown the co-existence of multiple insertion intermediates [8,10,14,15,16,17,18,19], the structure of the functional state of the T-domain responsible for the translocation of its N-terminus along with the catalytic domain remains unknown. The putative structural model representing the core in the post-translocated state (Figure 1b, bottom right structure) is based on the Open-Channel State (OCS) model [20] derived from conductivity measurements in planar bilayers [21,22,23] and is now confirmed by a combination of site-specific labeling and depth-dependent fluorescence quenching experiments [24].

To date, the only high-resolution structures of the diphtheria toxin are those obtained at neutral pH [26,27]. Here, we use the method of X-ray crystallography to gain insight into the early stages of acid-induced conformational changes in DT. For the first time, we report the structures obtained in a range of pH values from 7.0 to 5.0 (an example of the crystal at an acidic pH is presented in Figure S1). The observed structural changes are localized to the T-domain and are further characterized by comparison to new and previously published results for the isolated T-domain [11], obtained by molecular dynamics (MD) simulations, fluorescence and circular dichroism spectroscopy.

## 2. Results

### 2.1. Analysis of DT Structures Obtained at Varying pH

**Figure 2:**
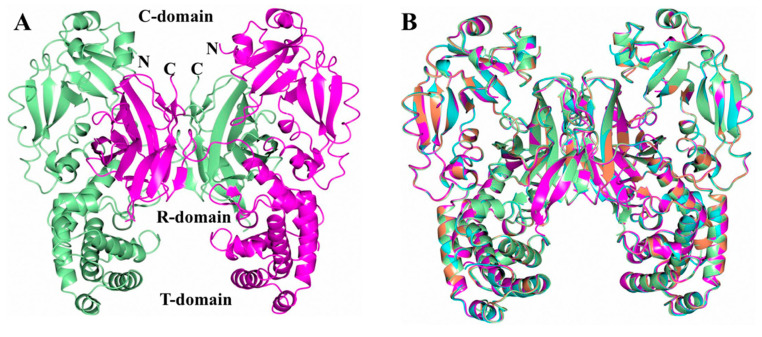
Structures of DT obtained at varying pH. (**A**) Structure of a DT-7.0 domain swapped dimer. Subunits A and B are colored green and magenta, respectively. (**B**) Superposition of DT-5.0 (green), DT-5.5 (coral), DT-6.0 (cyan), and DT-7.0 (magenta).

DT was crystallized under varying buffer conditions in an effort to determine if conformational changes, particularly in the T-domain, are observed with a decreasing pH. Since the crystallization conditions were identical except for the buffer, the crystals of DT-5.0, DT-5.5, DT-6.0, and DT-7.0 have similar unit cell dimensions and diffraction resolutions (Table 1). This represents a new crystal form of DT. However, the α-angle for DT-5.0 is approximately 4° larger than that of other crystals. The overall structure of this crystal form is similar to previously determined structures [26,27] and forms a domain-swapped dimer (Figure 2a). The structures of DT at various pH values were superimposed onto DT-5.0 using GESAMT in order to compare their similarities/differences. Overall, all of the structures are very similar (Figure 2b), with RMSD deviations between the C_α_ atoms of 0.34 Å (997 residues, DT-5.5), 0.38 Å (999 residues, DT-6.0), and 0.41 Å (1000 residues, DT-7.0). 

**Figure 3:**
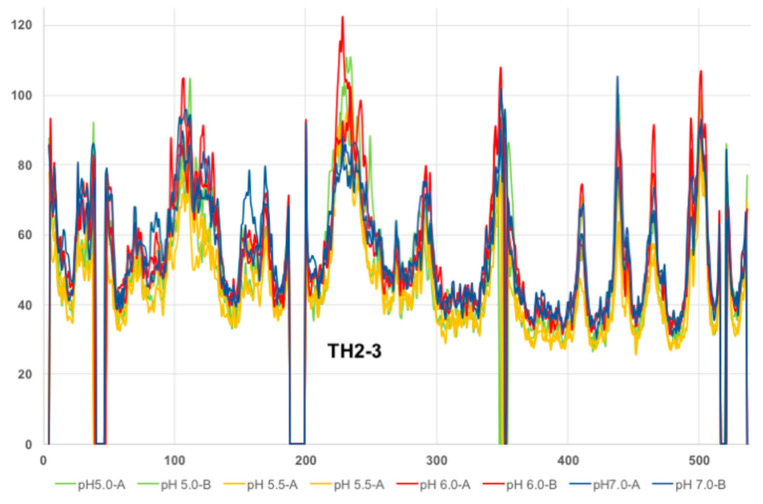
Plot of B-factors across the polypeptide for each subunit (A/B) of DT-7.0, DT-6.0, DT-5.5, and DT-5.0.

The mean main chain B-factors were plotted for all residues of each structure in an effort to analyze the flexibility across the polypeptide (Figure 3). One region that stands out is the TH2-TH3 region of the T-domain, which consistently displays large B-factors in each subunit of all structures. Interestingly, the residues between H223 to P234, which span TH2, appear to become more flexible at a lower pH. 

**Figure 4:**
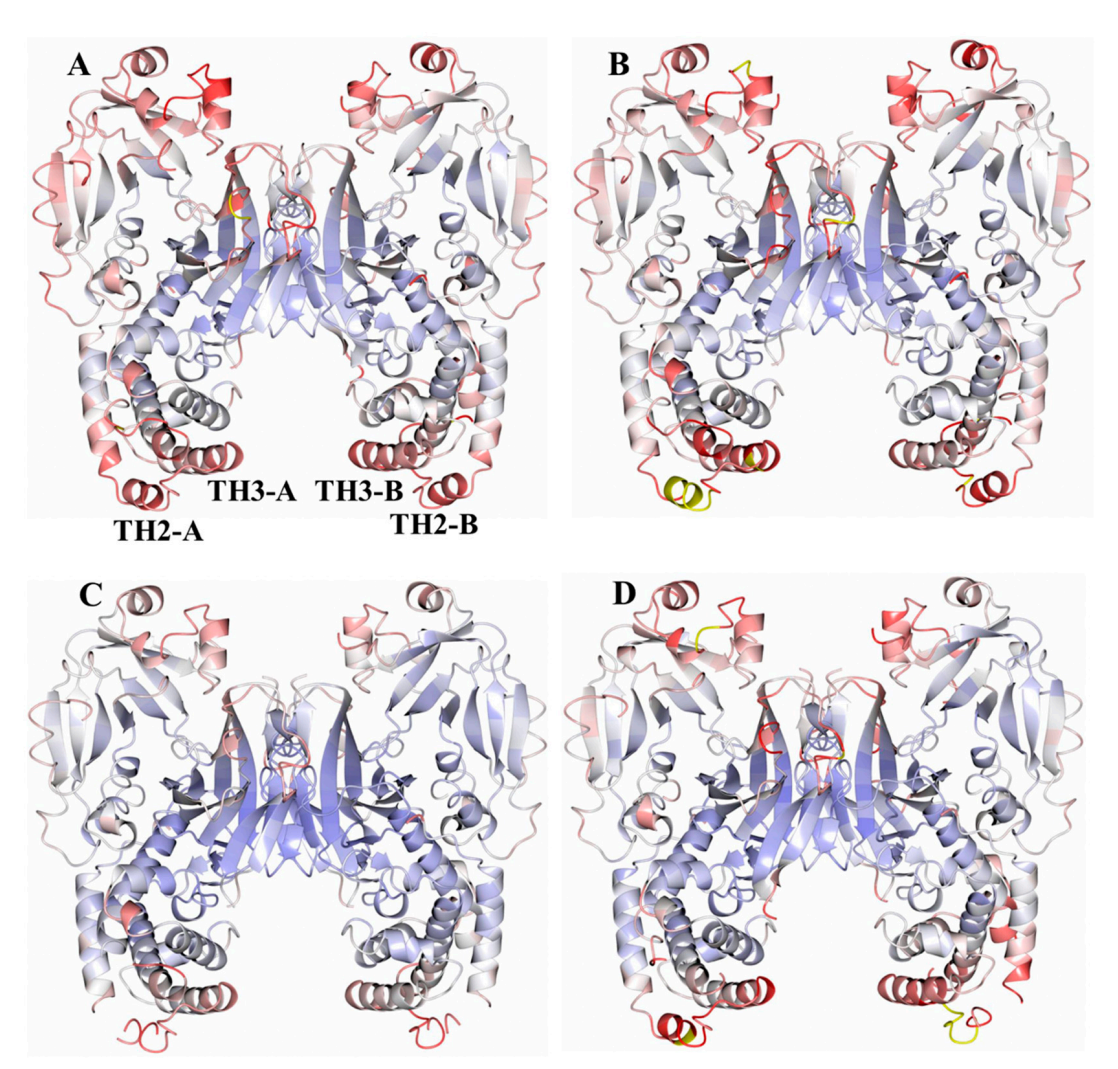
Structures of DT dimers colored by B-factor. (**A**) pH 7, (**B**) pH 6, (**C**) pH 5.5, (**D**) pH 5. The B-factor scale ranges are 0 Å^2^ (blue), 50 Å^2^ (white), and 100 Å^2^ (red). Residues with B-factors greater than 100 Å^2^ are colored yellow. The TH2 and TH3 helices are indicated in panel **A** for subunits A and B.

Analysis of the B-factors for each structure (Figure 4) shows ordered TH2 residues in DT-7.0 and slightly higher B-factors in this region for DT-6.0 in each subunit (TH2-A and TH2-B). This region in the DT-5.5 structure becomes more disordered and some of the residues could not be modeled due to weak or nonexistent electron density. In the DT-5.0 structure, the TH2 helix could be modelled in subunit A but not subunit B. It should be noted that this region does not form any crystal contacts that would cause conformational artifacts. When comparing the B-factors for multiple structures, one certainly needs to be cautious as each data set is unique. However, in this case we are analyzing crystals that are similar in their crystal forms and diffraction resolutions. As such, these structures suggest that the structural stability of TH2 is pH-dependent.

### 2.2. Comparison to pH-Dependent Refolding of Isolated T-Domain 

**Figure 5:**
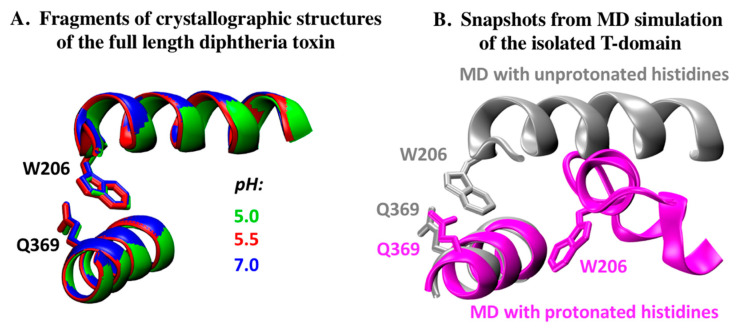
Comparison of packing of the first and last helices of the T-domain at different protonation states in the context of the crystallographic structure of the full-length DT and previously published MD simulations of the T-domain [11]. (**A**) Crystallographic structure of TH1 (with residue W206 highlighted), TH9 (with residue Q369 highlighted), and W206 (N-terminal helix TH1) exhibits no variation at pH 7.0 (blue), 5.5 (red), or 5.0 (green). (**B**) MD simulations for unprotonated T-domain demonstrate the close packing of N-terminal TH1 and C-terminal TH9 of the T-domain (grey). Protonation of the six histidine residues results in the collapse of the TH1 (magenta) [11]. The resulting separation of the N- and C-terminal segments of the T-domain changes the proximity of residues Q369 and W206, which can be studied by means of fluorescence spectroscopy (see Figure 6).

Our earlier MD study of the isolated T-domain revealed that histidine protonation, while not accompanied by the loss of structural compactness of the protein, nevertheless drives substantial molecular rearrangements characterized by the partial loss of secondary structures due to the unfolding of helices TH1 and TH2 and the loss of close contact between the C- and N-terminal segments [11]. While the high-resolution structures at a low pH presented here confirm the unfolding of TH2 (Figure 3 and Figure 4), no changes in the structure of TH1 nor in its proximity to TH1 at pH 7.0–5.0 have been observed (Figure 5a and Figure S2). This warrants a closer look at the computational and experimental characterization of the structure of the isolated T-domain presented below. 

The MD simulations of the isolated T-domain revealed that, in the neutral pH simulation, the protein retained its globular structure in a natively folded state. This was evident from the absence of significant structural changes and the low values (about 1.9 Å) of the average root-mean square deviation of the Cα atoms in TH1–TH9 from their crystallographic positions [11]. In stark contrast to the neutral pH simulation, large structural changes were observed in the protein at low pHs. Figure 5b shows the comparison of the folding of the TH1 segment of the protein estimated by MD simulations with unprotonated and protonated histidines.

**Figure 6:**
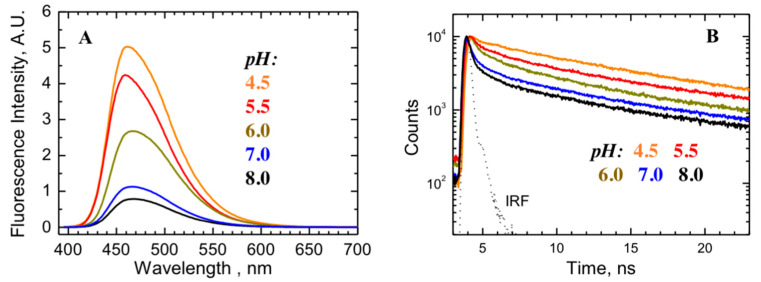
Fluorescence-based proximity measurements between bimane probe attached to a single cysteine mutant at position 369 (Q369C mutant) and residue W206. The short-range aromatic quenching of bimane steady-state (**A**) and time-resolved fluorescence (**B**) indicates close proximity in the folded structure at neutral pH. The Instrument Response Function (IRF) is shown as a dotted line. The reduction in quenching observed at an acidic pH indicates the loss of close packing between the N- and C-termini of the T-domain, also observed in the MD simulation (see Figure 5b and [11]).

At a mildly acidic pH, the T-domain undergoes partial unfolding and the resulting loss of ellipticity signal from the helical structure, as measured by circular dichroism spectroscopy [11]. The thermodynamic stability of the T-domain is also substantially reduced already at pH 6, before any of the complicating effects of acid-caused precipitation can be noticed [19]. We validated the MD observations that the conformational reorganization of the T-domain upon histidine’s protonation results in an increase in the distance between Q369 in the C-terminus of TH9 and W206 in the N-terminus of TH1 (Figure 5b). We applied the fluorescence quenching technique, which is sensitive to changes in this distance scale, by replacing Q369 with a cysteine and labeling it with bimane dye. The fluorescence of bimane is known to be strongly quenched by aromatic residues (e.g., W206) in a short-distance range (less than 10 Å) [34,35]. Figure 6b–c, show the steady-state fluorescence spectra and lifetime decay measurements of bimane-labeled T-domain at pH 8, exhibiting low intensity and highly quenched kinetics with a pronounced short-lived component (Figure 6b–c, black curves), indicating a close proximity of W206 and a bimane probe. The quenching is decreased at pH 4.5 (Figure 6a,b, orange curves), consistent with the loss of contact between TH1 and TH9 observed in the MD simulation (Figure 5b, magenta). At intermediate pHs of 6.0–5.5, the lifetime kinetics can be represented by the mixture of quenched and unquenched fluorescence species, which implies the coexistence of folded and unfolded states in solution (our interpretation of the bimane fluorescence results is supported by the substantially reduced quenching (and hence reduced pH-dependent recovery) observed when the probe is attached one helical turn further along TH9 in the N366C mutant (Figure S3)). 

## 3. Discussion

Since the first structures of the diphtheria toxin were published in the early 1990s [26,27], our understanding of the many aspects of the cellular entry of the toxin, illustrated in Figure 1a, has dramatically progressed. Nevertheless, full understanding of atomistic details of the membrane translocation process remains elusive (Figure 1b). In the past few decades, many research groups had contributed to deciphering of the action of the T-domain of the toxin [8,10,14,15,16,17,18,19] and the involvement of the protonation of various titratable residues in its conformational switching [10,11,12,13,18,19,36,37,38,39]. The emerging picture is that of a complex multistep process, characterized by overlapping pH-dependent transitions occurring both in solution and on the membrane interface [10,25]. Here, we have used X-ray crystallography along with fluorescence spectroscopy to characterize early conformational changes occurring in solution at a mildly acidic pH.

The overall structure of the DT at pH 7, featuring a domain-swapped dimer (Figure 2a), corresponds well to that previously published [26]. The set of the structures generated for mildly acidic pH values of 6, 5.5, and 5 retain essentially the same conformations (Figure 2b), with the hotspots of partial unfolding highlighted by the elevated B-factors (Figure 3 and Figure 4). The observed loss of the helical structure in helices TH2 and TH3 of the T-domain are of a special interest to us, since these regions are expected to unfold upon acidification based on the previous results of MD simulations [11] and Hydrogen-Deuterium Exchange Mass-Spectrometry for the isolated T-domain [36]. In contrast, the long TH1, which is also expected to refold based on the latter studies, remains unchanged in the crystal structure even at pH 5 (Figure 5). In general, such a result can be explained in two ways—either by limitations imposed by the crystal or by the different folding behavior of the isolated T-domain as compared to that in the full-length protein. We suspect that, to some degree, both factors may be involved here. The only crystal contact that TH1 makes is an H-bond between E218 and S535 from the neighboring molecules related by translational symmetry (not shown). While this is unlikely to be sufficient for helix stabilization, the overall proximity of the neighboring DT molecule might impose some limitations on what conformations TH1 can adopt, reducing the entropic component driving the refolding at an acidic pH.

The more likely explanation of the variation in the pH-dependent behavior of the TH1 helix (compare Figure 5a,b) involves the somewhat different refolding pattern of the isolated T-domain. MD simulations mimicking mildly acidic conditions by protonating the six histidine residues in the T-domain indicate that both the TH1 and TH2 segments lose their secondary structure and that the close packing of the C- and N-terminal segments of the T-domain is lost. Both of these conclusions are supported by both new and previously published spectroscopic experiments with the isolated T-domain shown in Figure 6. The pronounced decrease in negative ellipticity observed in the published circular dichroism pH titration is consistent with the substantial loss of the helical content [11]. The bimane experiment shows that acidification also causes a progressive increase in fluorescence intensity (Figure 6a) and an increase in the fluorescence lifetime of the probe attached at residue 369 in a Q369C mutant (Figure 6b). These changes are caused by the relief of the quenching of the bimane fluorescence with the aromatic side-chain of W206, thus indicating the displacement of the terminal segments containing the probe and the quencher from each other at an acidic pH. While such a displacement can be expected in the context of the isolated T-domain, it would be less likely in the context of full-length DT, where the T-domain is flanked on both sides by the C- and R-domains. This question will be further addressed in future computational and experimental studies involving the entire protein.

## 4. Conclusions

For the first time, we present the high-resolution structure of the diphtheria toxin at a mildly acidic pH (5–6) and compare it to the structure at neutral pH (7). We demonstrate that neither catalytic nor receptor-binding domains change their structure upon this acidification, while the T-domain undergoes a conformational change that results in the unfolding of TH2–3 helices. Surprisingly, the TH1 helix maintains its conformation in the crystal of the full-length toxin even at pH 5. This contrasts with the evidence from the new and previously published data, obtained by spectroscopic measurements and molecular dynamics computer simulations, that indicate the refolding of TH1 upon the acidification of the isolated T-domain. The overall results imply that the membrane interactions of the T-domain are critical in ensuring the proper conformational changes required for the preparation of the diphtheria toxin for cellular entry.

## 5. Materials and Methods 

### 5.1. Materials

Bovine thrombin was from Fisher Scientific (Pittsburgh, PA, USA). 

### 5.2. Preparation of the T-Domain and Full-Length DT

Both the full-length DT and the T-domain were prepared as described in [10]. Briefly, the protein expression was examined in BL21 DE3pLys E. coli cells, recombinant protein synthesis was induced by the addition of 0.8 mM of IPTG at OD600 = 0.5, after which cells were grown at 25 °C overnight. Purification included affine chromatography on Ni-NTA resin from Qiagen (Valencia, CA, USA) and gel-filtration on a Sepharose 12 1 × 30 cm column from GE Healthcare (Chicago, IL, USA) in PBS buffer containing 0.1 mM of EDTA. The purity of the preparations obtained was analyzed by SDS PAGE. For the determination of the protein concentration, we used a molar extinction coefficient of 17,000 M^−1^cm^−1^ at 278 nm for the T-domain and 49,600 M^−1^cm^−1^ for full-length diphtheria toxin (DT). As a template for the expression of full-length DT, we used E148S/C201S mutant, which is known to have reduced cytotoxicity [40].

Labeling with bimane was performed using a standard procedure for thiol-reactive derivatives [41]. We used a monobromobimane derivative (Invitrogen, Eugene, OR, USA). Typically, 1 mg of the dye was dissolved in 50 µL of DMFA and added drop-wise to the protein solution in PBS (pH 7.4) containing 0.1 mM of EDTA. The reaction mixture was incubated for two hours at room temperature or overnight at 4 °C. Unreacted dye was removed by gel filtration chromatography on a HiPrep 26/10 desalting column ran on an FPLC AKTA Purifier system (GE Healthcare, Chicago, IL, USA), followed by at least five consecutive centrifugations using a Microcon YM-10 concentrator, until the solution coming through the concentrator did not contain any dye, as assayed by absorbance spectroscopy.

### 5.3. Crystallization and Data Collection

Purified diphtheria toxin (DT) was concentrated to 9.2 mg/mL (WT) in 50 mM of disodium phosphate at pH 8.0 for crystallization screening. All the crystallization experiments were set up using an NT8 drop setting robot (Formulatrix Inc., Bedford, MA, USA) and UVXPO MRC (Molecular Dimensions, Maumee, OH, USA) sitting drop vapor diffusion plates at 18 °C. A total of 100 nL of protein and 100 nL of crystallization solution were dispensed and equilibrated against 50 uL of the latter. Plate-shaped crystals of native DT were obtained from 10% (w/v) PEG 10K, 100 mM of magnesium acetate containing the following buffers and pH values: 100 mM of MES at pH 5.0, (DT-5.0), 100 mM of MES at pH 5.5 (DT-5.5), 100 mM of MES at pH 6.0 (DT-6.0), and 100 mM of HEPES at pH 7.0 (DT-7.0). Crystals were transferred to cryoprotectant solution composed of 80% crystallization solution and 20% (v/v) PEG 200, harvested with a cryoloop and stored in liquid nitrogen. X-ray diffraction data were collected at the Advanced Photon Source beamline 17-ID using a Dectris Pilatus 6M pixel array detector.

### 5.4. Structure Solution and Refinement

Intensities were integrated using XDS [42,43] via AutoPROC [44], and the Laue class analysis and data scaling were performed with Aimless [28] which indicated that the crystals belonged the triclinic space group P1. The Matthews coefficient [45] for all the data indicated that a non-crystallographic dimer was present in the asymmetric unit. Structure solution for DT-WT-pH5 was examined by molecular replacement with Phaser [46] using a previously determined DT structure as the search model (PDB 1DDT). The final model of DT-pH5 was used as the search model for molecular replacement against the other datasets. Model refinement and manual model building were conducted with Phenix and Coot [47] respectively. Disordered side chains were truncated to the point for which electron density could be observed. Structure validation was conducted with MolProbity [48] and figures were prepared using the CCP4MG package [49]. Structure superposition was carried out with GESAMT [50]. Crystallographic data are provided in Table 1.

### 5.5. Fluorescence Measurements

Fluorescence was measured using an SPEX Flurolog FL 3–22 steady-state fluorescence spectrometer (Jobin Yvon, Edison, NJ, USA) equipped with double grating excitation and emission monochromators. The measurements were made at 25 °C in 2 × 10 mm cuvettes oriented perpendicular to the excitation beam. For the bimane fluorescence measurement, the excitation emission wavelength was 380 nm and the emission spectra were recorded between 395 and 700 nm using excitation and emission spectral slits of 2 and 4 nm, respectively. Solution acidification was achieved by the addition of small amounts of 2.5 M acetic buffer. All the spectra were recorded after 30 min of incubation to ensure the equilibrium of the sample.

The fluorescence lifetime kinetics of bimane-labeled T-domain were measured with a time-resolved fluorescence spectrometer FluoTime 200 (PicoQuant, Berlin, Germany) using a standard time-correlated single-photon counting scheme. Samples were excited at 373 nm by a sub-nanosecond pulsed diode laser LDH 375 (PicoQuant, Berlin, Germany) with a repetition rate of 10 MHz. Fluorescence emission was detected at 480 nm, selected by a Sciencetech Model 9030 monochromator, using a PMA-182 photomultiplier. The fluorescence intensity decay was analyzed using the FluoFit version 2.3 iterative-fitting software based on the Marquardt algorithm (PicoQuant, Berlin, Germany).

## Figures and Tables

**Table 1:** Crystallographic data for diphtheria toxin structures (continues next page).

	DT-5.0	DT-5.5	DT-6.0	DT-7.0
**Data Collection**				
Unit-cell parameters (Å, °)	*a* = 69.04 *b* = 69.16 *c* = 73.38 *α* = 122.1 *β* = 93.7 *γ* = 97.9	*a* = 69.55 *b* = 69.67 *c* = 73.40 *α* = 117.6 *β* = 93.5 *γ* = 98.1	*a* = 69.44 *b* = 69.61 *c* = 73.12 *α* = 117.9 *β* = 93.9 *γ* = 97.9	*a* = 69.38 *b* = 69.64 *c* = 73.15 *α* = 117.5 *β* = 93.3 *γ* = 98.3
Space group	*P*1	*P*1	*P*1	*P*1
Resolution (Å) ^1^	49.59-2.05	46.77-2.05	46.57-2.10	46.74-2.30
Wavelength (Å)	1.0000	1.0000	1.0000	1.0000
Temperature (K)	100	100	100	100
Observed reflections	239,575	253,521	236,012	183,332
Unique reflections	68,617	72,784	67,346	51,695
<I/ (I)> ^1^	10.0 (1.7)	9.8 (1.7)	10.7 (1.8)	9.7 (1.8)
Completeness (%) ^1^	97.3 (96.6)	96.9 (96.8)	97.4 (96.9)	97.7 (97.3)
Multiplicity ^1^	3.5 (3.5)	3.5 (3.5)	3.5 (3.5)	3.5 (3.6)
R_merge_ (%) ^1,2^	6.2 (79.2)	6.6 (79.8)	5.7 (69.6)	7.3 (75.4)
R_meas_ (%) ^1,4^	7.4 (93.6)	8.2 (93.9)	7.3 (83.1)	8.6 (88.5)
R_pim_ (%) ^1,4^	3.9 (49.3)	4.2 (49.8)	3.6 (43.9)	4.5 (46.0)
CC_1/2_^1^	0.998 (0.681)	0.998 (0.656)	0.998 (0.753)	0.997 (0.698)
**Refinement**				
Resolution (Å) ^1^	36.61-2.05	35.03-2.05	36.43-2.10	32.72-2.30
Reflections (working/test) ^1^	65,164/3424	69,202/3554	64,135/3175	49,240/2428
*R*_factor_ / *R*_free_ (%) ^1,3^	19.7/25.0	18.3/24.3	21.0/25.8	18.5/24.1
No. of atoms (Protein/Water)	7444/321	7401/353	7491/227	7591/201
**Model Quality**				
R.M.S deviations				
Bond lengths (Å)	0.009	0.009	0.010	0.009
Bond angles (°)	0.915	0.905	0.941	0.959
Mean *B*-factor (Å^2^)				
All Atoms	50.3	46.6	56.0	56.3
Protein	50.5	46.7	56.3	56.5
Water	46.0	44.4	48.4	48.9
Coordinate error (maximum likelihood) (Å)	0.28	0.25	0.28	0.32
Ramachandran Plot				
Most favored (%)	96.1	97.1	95.6	96.2
Additionally allowed (%)	3.5	2.4	3.6	2.9

Values in parenthesis are for the highest resolution shell. ^1^ *R*_merge_ = Σ_hkl_Σ_i_|*I*_i_(hkl) − < I(hkl) > |/Σ_hkl_Σ_i_ *I*_i_(hkl), where *I*_i_(hkl) is the intensity measured for the *i*_th_ reflection and < I(hkl) > is the average intensity of all reflections with indices hkl. ^2^ *R*_factor_ = Σ_hkl_||*F*_obs_(hkl)| − |*F*_calc_(hkl)||/Σ_hkl_|*F*_obs_(hkl)|; *R*_free_ is calculated in an identical manner using 5% of randomly selected reflections that were not included in the refinement. ^3^ *R*_meas_ = redundancy independent (multiplicity-weighted) *R*_merge_ [28,29]. *R*_pim_ = precision indicating (multiplicity-weighted) *R*_merge_ [30,31]. ^4^ CC1/2 is the correlation coefficient of the mean intensities between two random half-sets of data [32,33].

## Supplementary Materials

The following are available online at https://www.mdpi.com/2072-6651/12/11/704/s1: Figure S1: Crystals of diphtheria toxin; Figure S2: Structure of diphtheria toxin dimer at pH 7 with T-domain helices highlighted in blue (TH1), green (TH2–3) and red (TH9); Figure S3: Comparison of the steady-state (A,C) and time-resolved (B,D) fluorescence quenching of bimane probe attached to attached to single cysteine mutants Q369C (A,B) and N366C (C,D) mutant.
