# Supplementary material for: Structure of the Diphtheria Toxin at Acidic pH: Implications for the Conformational Switching of the Translocation Domain
Source: Toxins (Basel). 2020 Nov 7;12(11):704. doi: 10.3390/toxins12110704 (PMC7695028; doi:10.3390/toxins12110704)
Supplement: Supplementary file 1 [file toxins-12-00704-s001.pdf]

Supplementary Materials: Structure of the Diphtheria Toxin at Acidic pH: Implications for the Conformational Switching of the Translocation Domain

Mykola V. Rodnin, Maithri M. Kashipathy, Alexander Kyrychenko, Kevin P. Battaile, Scott Lovell and Alexey S. Ladokhin

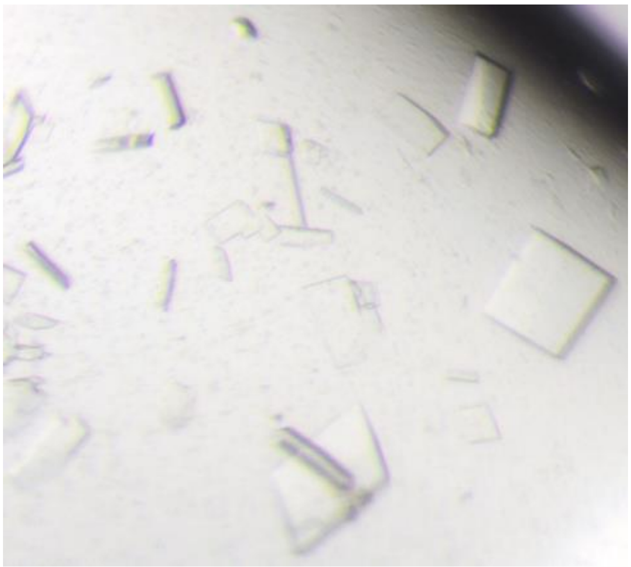

Figure S1. Crystals of diphtheria toxin. 10% (w/v) PEG 10K, 100 mM MES pH 5.0, 100 mM magnesium acetate.

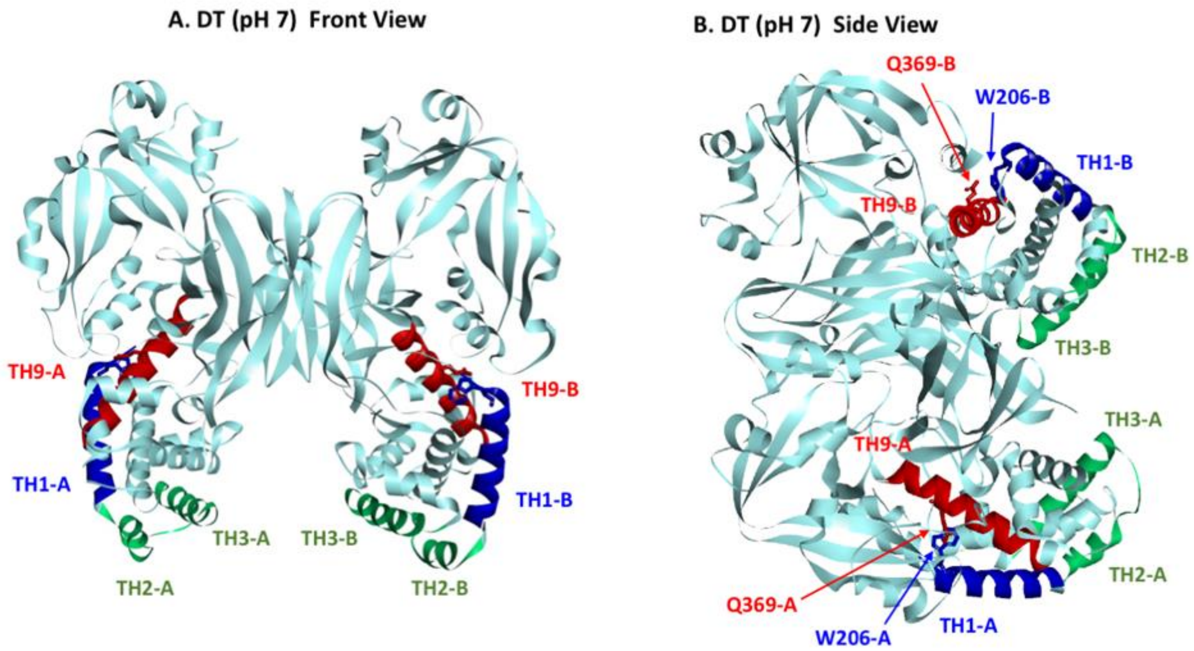

Figure S2. Structure of Diphtheria Toxin dimer at pH 7 with T-domain helices highlighted in blue (TH1), green (TH2–3) and red (TH9). (**A**) The orientation corresponds to that in Figure 2 and 4. (**B**) The orientation approximately corresponds to that of the fragment in Figure 5. Residues Q369 (TH9) and W206 (TH1) are highlighted by stick representation in red and blue, respectively.

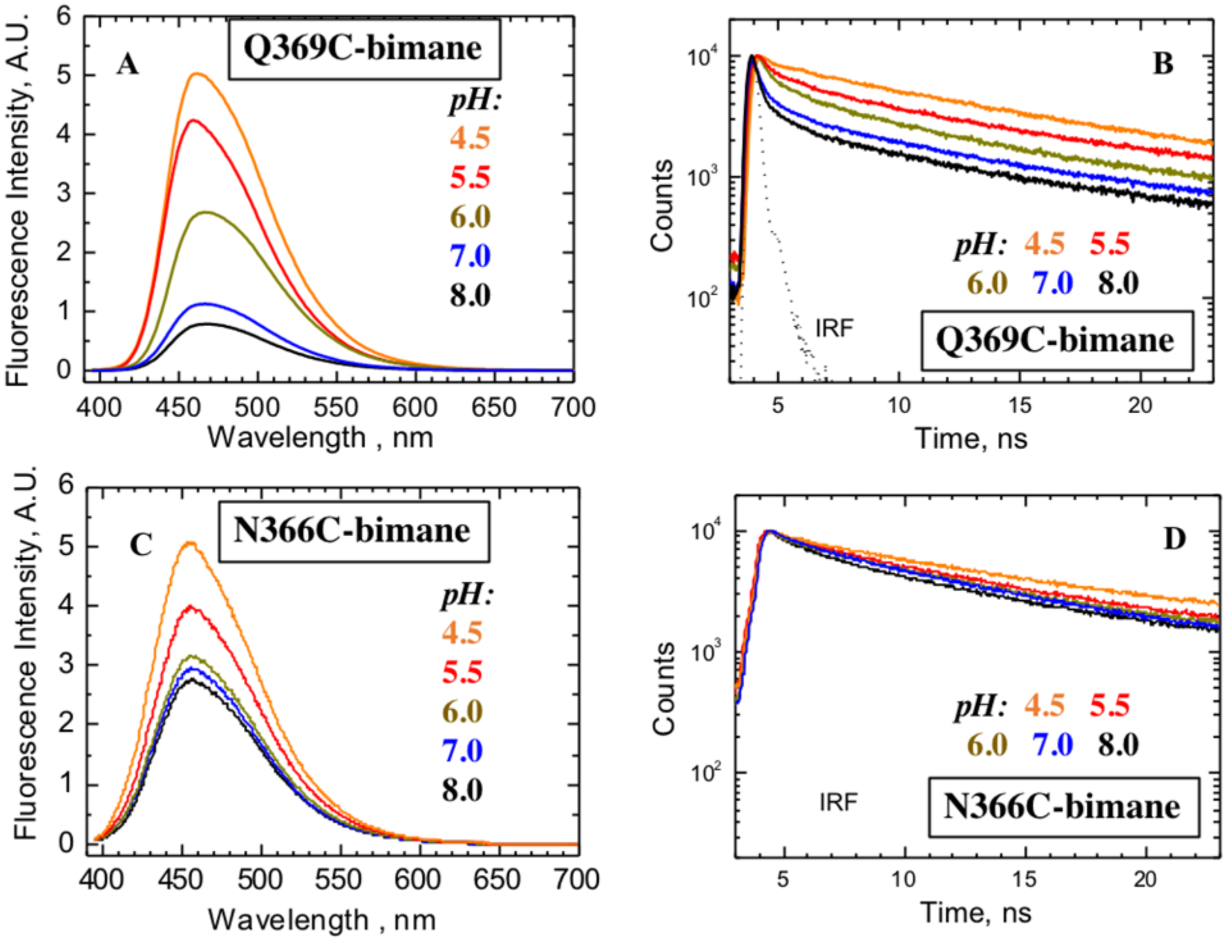

Figure S3. Comparison of the steady-state (**A**,**C**) and time-resolved (**B**,**D**) fluorescence quenching of bimane probe attached to attached to single cysteine mutants Q369C (**A**,**B**) and N366C (**C**,**D**) mutant. The data for Q369C-Bimane are the same as those shown in the main text (Figure 6**A**,**B**) and are presented here for the ease of comparison. The quenching effects that are strongly pronounced at neutral pH for Q369C, are much smaller for N366C. This correlates to their proximity of the bimane probe to W206 (quencher) in the folded structure.
